# Hypoxia potentiates the capacity of melanoma cells to evade cisplatin and doxorubicin cytotoxicity via glycolytic shift

**DOI:** 10.1002/2211-5463.12830

**Published:** 2020-04-14

**Authors:** Ming Zhuo, Falih M. Gorgun, Douglas S. Tyler, Ella W. Englander

**Affiliations:** ^1^ Department of Surgery University of Texas Medical Branch Galveston TX USA

**Keywords:** cisplatin, doxorubicin, glycolysis, hypoxia, melanoma, mitochondrial respiration

## Abstract

The hypoxic environment within solid tumors impedes the efficacy of chemotherapeutic treatments. Here, we demonstrate that hypoxia augments the capacity of melanoma cells to withstand cisplatin and doxorubicin cytotoxicity. We show that B16F10 cells derived from spontaneously formed melanoma and YUMM1.7 cells, engineered to recapitulate human‐relevant melanoma driver mutations, profoundly differ in their vulnerabilities to cisplatin and doxorubicin. The differences are manifested in magnitude of proliferative arrest and cell death rates, extent of mtDNA depletion, and impairment of mitochondrial respiration. In both models, cytotoxicity is mitigated by hypoxia, which augments glycolytic metabolism. Collectively, the findings implicate metabolic reprogramming in drug evasion and suggest that melanoma tumors with distinct genetic makeup may have differential drug vulnerabilities, highlighting the importance of precision anticancer treatments.

## Graphical abstract

Cancer cells have robust drug evasion capabilities. We show that the genetically distinct B16F10 and YUMM1.7 melanoma models have differential drug vulnerabilities, suggesting that tumors arising from the same tissue may acquire unique weaknesses and differentially respond to therapy. Notwithstanding, in hypoxia, both cell models exploit metabolic reprogramming for drug evasion that could explain chemotherapy failures in arresting tumor growth.

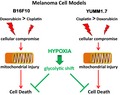

**Abbreviations**
2DG: 2‐deoxy‐d‐glucose
FCCP: carbonyl cyanide *p*‐trifluoromethoxyphenylhydrazone
mtDNA: mitochondrial DNA
Tfam: mitochondrial transcription factor A
NER: nucleotide excision repair
OCR: oxygen consumption rates
SRC: spare respiratory capacity
Top2: topoisomerase 2

The importance of precisely tailored cancer treatment strategies to achieving satisfactory outcomes is increasingly evident [1]. Here, we studied responses of two mouse melanoma cell models to the widely used chemotherapeutic drugs, cisplatin and doxorubicin, which block cancer cell proliferation via induction of different types of DNA damage. Cisplatin, a platinum‐based drug, forms crosslinks with both nuclear [2] and mitochondrial DNA (mtDNA) [3]. However, while DNA:cisplatin crosslinks in nuclear DNA are repairable by the ubiquitous nucleotide excision repair (NER) pathway, NER is absent from the mitochondrial compartment [4, 5], where cisplatin:mtDNA crosslinks are likely to persist and contribute to mitochondrial dysfunction. On the other hand, doxorubicin that belongs to the anthracycline antibiotics family damages DNA by intercalation between DNA base pairs and formation of stable complexes with topoisomerase 2 (Top2) isoforms, thereby preventing relief of topological stress and blocking replication and other DNA transactions [6, 7]. We used cisplatin and doxorubicin to challenge two melanoma cell lines, the B16F10 cells that were derived from a spontaneously generated mouse melanoma tumor [8] and the recently established YUMM1.7 (Yale University Mouse Melanoma) cell line engineered to recapitulate three human‐relevant melanoma driver mutations [9]; the Braf^V600E^ mutation that activates proliferative signaling and two tumor suppressor loss‐of‐function mutations Pten^−/−^ and Cdkn2a^−/−^ [9, 10, 11, 12]. In contrast to YUMM1.7, B16F10 cells lack the Braf^V600E^ activating mutation while similarly carrying mutated Pten and Cdkna2 genes [13]. While it is difficult to predict how genetic makeup might shape the melanoma cell phenotype, here, we show that B16F10 and YUMM1.7 cells have distinctive properties, markedly differing in cell morphology, glycolytic gene expression profiles, mitochondrial contents, and vulnerabilities to DNA damaging drugs. We demonstrate that similarly to other types of cancer cells [1], B16F10 and YUMM1.7 cells exhibit metabolic plasticity and under hypoxia readily shift to glycolytic metabolism. Importantly, in agreement with salient ability of cancer cells to evade toxicity of chemotherapeutic drugs in hypoxic tumors [14], B16F10 and YUMM1.7 cell lines better withstand exposures to DNA damaging drugs under hypoxic conditions.

We expect that collectively, our findings alert to the individual differences among tumors derived from the same type of tissue and to the critical importance of informed precisely tailored design of chemotherapeutic treatments in achieving desired outcomes.

## Materials and methods

### Cell cultures and treatments

All mouse cell lines were purchased from ATCC and inspected for mycoplasma: YUMM1.7 melanoma cell line [9] (ATCC CRL‐3362) was cultured in DMEM/F12 medium (Invitrogen #11320033, Waltham, MA, USA) with 10% FBS (ATCC #30‐2020), 1% nonessential amino acid (Gibco # 11440‐076, Gaithersburg, MD, USA), 1% penicillin/streptomycin, and maintained at confluence below 85%. The B16F10 cell line (ATCC CRL‐6475) [15] was cultured in DMEM (Invitrogen # 11965092) with 10% FBS, and NIH3T3 embryonic fibroblast cell line (ATCC CRL‐1658) was cultured in DMEM with 10% bovine calf serum (ATCC #30‐2030). Hypoxia treatments were done as described [16]; briefly, cultures were placed in a gas‐tight modular incubator chamber (Billups Rothenberg, Del Mar, CA) and flushed for 3 min with 5% CO_2_ balanced with 95% nitrogen at a flow rate of 50 L·min^−1^. The oxygen content in the chamber was 0.8 ± 0.2% measured by the oxygen pen 800047 (Sper Scientific, Scottsdale, AZ, USA) with water container placed in the chamber to maintain a humidified environment. Cisplatin (Sigma, Perkasie, PA, USA) at concentrations ranging from 10‐20 µm or doxorubicin (NDC 0069‐3031‐20 Pfizer) at 1 and 2 µm was added for 14‐h incubations under normoxic or hypoxic conditions. Trypan blue exclusion was used to quantify living and dead cells. Cells were cultured in 24‐well plates; upon the termination of treatments, cells were collected by trypsinization, spun down at 500 g/3 min, resuspended in 200 µL culture medium, and 10 µL of cell suspension mixed with 10 µL 0.4% trypan blue (Cellgro, Manassas, VA, USA). Living and dead (blue) cells were counted in triplicates using hemacytometer and a brightfield microscope. Data from three sets of independent experiments were compiled and IC50 values were calculated with prism 7.0a software (GraphPad, La Jolla, CA, USA) by using a nonlinear regression with a variable slope fit function.

### Real‐Time qPCR determination of mRNA levels and mtDNA copy number

Total RNA was isolated using RNeasy plus mini kit (Qiagen, Valencia, CA, USA). Reverse transcription was done with iScript RT supermix (Bio‐Rad, Hercules, CA, USA) and RT‐qPCR analyses done with CFX96 Real‐Time System (Bio‐Rad) using primers provided in Table 1. The 18s gene was used as reference for calculation of relative expression levels. PCRs were with SSO FAST EvaGreen® supermix (Bio‐Rad) and cycling program: 95°C 2 min, 40 cycles of 2‐step incubation, first at 95°C 5 s then at 55°C 15 s followed by melting curve analysis. Expression levels relative to control for a given gene were calculated using the formula: −ΔΔCt=[(CT ^gene of interest^ − CT ^internal control^) sample − (CT ^gene of interest^ − CT ^internal control^) control]. For comparison of expression levels among cell lines, relative gene expression was calculated using the formula: relative expression = 2^[‐(CT gene of interest− CT internal control)]^ × 10 000 [17, 18].

**Table 1:** IDs and sequences of mouse primers

Gene Symbol	Forward	Reverse	Accession number	Gene name
mtDNA targets				
Cox 1	cagaccgcaacctaaacaca	ttctgggtgcccaaagaat	JF286601, *Mus musculus* strain C57BL/6 mitochondrion complete genome	
Cox3	caattacatgagctcatcatagc	ccatggaatccagtagcca	JF286601, *Mus musculus* strain C57BL/6 mitochondrion complete genome	
Nd 1	catgatctaggaggctgctgac	cgtttaccttctataaggctatga	JF286601, *Mus musculus* strain C57BL/6 mitochondrion complete genome	
nuclear targets				
18s	gtaacccgttgaaccccatt	ccatccaatcggtagtagcg	NR_003278.3	*Mus musculus* 18S ribosomal RNA
B2m	atccaaatgctgaagaacgg	atcagtctcagtgggggtga	NM_009735	Beta‐2 microglobulin
Hk2	cagtgaacctttgactcatctca	gatgacaaaacgctcactagacc	NM_013820.3	Hexokinase 2
Gpi1	ccctctttataatcgcctcca	gaaaccactcctttgctgtctc	NM_008155.3	Glucose phosphate isomerase 1
Aldo A	tgggaagaaggagaacctga	gacaagcgaggctgttgg	NM_007438.4	Aldolase A, fructose‐bisphosphate
Tpi 1	aaaccaaggtcatcgcaga	cccggagcttctcgtgta	NM_009415.2	Triosephosphate isomerase 1
Pgk 1	tacctgctggctggatgg	cacagcctcggcatatttct	NM_008828.2	Phosphoglycerate kinase 1
Pgam1	cctcatggtgatttttaaccctaa	aagattgatcccaaccttctagg	NM_023418.2	Phosphoglycerate mutase 1
Pkm2	aagggggactaccctctgg	cctcgaatagctgcaagtgg	NM_011099.2	Pyruvate kinase 2
Ldh A	ggcactgacgcagacaag	tgatcacctcgtaggcactg	NM_010699.2	Lactate dehydrogenase A

Mitochondrial DNA copy number determination: Total DNA was isolated from 10^6^ cells cultured to ~ 60% confluence in 6‐well plates, using easy DNA isolation kit (Life Technologies); RNA was digested with RNase A (40 μg/mL/30 min/37°C). RT‐qPCRs were assembled with 10 ng total DNA and SSO FAST EvaGreen® supermix (Bio‐Rad). The nuclear beta‐2‐microglobulin (B2m) gene was used as reference. A 180‐nucleotide segment of mitochondria‐encoded cytochrome oxidase subunit 3 (Cox3) was amplified, and assays were confirmed by amplification of the mitochondria‐encoded genes, cytochrome c oxidase subunit 1 (Cox1), and Nd1. mtDNA copy number was calculated using the formula: mtDNA copy number = 2 × 2^(CTCoxIII−CTB2M)^ [19]. Mean ± SEM values for mtDNA copy number were calculated from 3 to 4 sets of biological experiments.

### Measurement of lactate in culture medium

Lactate concentration in culture medium was determined using L‐lactate kit (#1200014002, Eton Bio, San Diego, CA, USA) as described [20]. After debris removal by 10 000 g/5 min spin, medium was diluted 1: 10 and 50 μL aliquots placed in 96‐well plates, mixed with 50 μL assay reagent, incubated 30 min/37 °C, and terminated with 0.5 m acetate. O.D. was measured at 450 nm (TECAN FL200 plate reader). Lactate concentrations were calculated from a standard curve using magellan™ software (Tecan, Morrisville, NC, USA). Data are presented as mean ± SEM of four biological experiments.

### Immunofluorescence and immunocytochemistry

B16F10 and YUMM1.7 cells were seeded on coverslips in 24‐well plates, treated as indicated and fixed in 4% paraformaldehyde [16, 21]. Cells were permeabilized with 0.1% Triton X‐100/0.1% sodium citrate in PBS and blocked with 3% BSA (w/v)/1% donkey serum (v/v) in PBS followed by mouse anti‐Cox1 (1: 1500) antibody (Invitrogen‐459600). Coverslips were washed 3x with 1% BSA and incubated 45 min with goat‐anti mouse 594 AlexaFluor (1: 2500) secondary antibody, mounted with Prolong® Gold Anti‐fade with DAPI, and viewed with 40x objective on Olympus IX71 with QIC‐F‐M‐12‐C cooled camera (QImaging, Surrey, BC, USA) and qcapture pro (QImaging) software. For immunocytochemistry, similarly processed coverslips were incubated with rabbit anti‐Tfam primary antibody (1: 500 Genetex‐GTX103231). After three washes with 1% BSA/PBS, ImmPRESS‐HRP anti‐rabbit IgG (Vector MP‐7451) was applied for 30 min, washed, developed by DAB (Immpact DAB kit, Vector SK‐4105), and counterstained by hematoxylin (TA060MH, Thermo Scientific, Millersburg, PA, USA). Images were captured with brightfield 60x oil objective (E600 microscope system, Nikon). Chromogen intensity was quantified using the reciprocal intensity approach [22] and imagej software. Briefly, free hand tool was used to select an absolute white area and measure the base level intensity followed by outlining a selected cell and measuring its intensity level. Reciprocal intensity of each given cell was calculated using the formula: reciprocal intensity (*r*) = baseline intensity − target cell staining intensity. For each treatment, 30‐50 cells from randomly selected fields were measured. Data are presented as mean ± SEM of three biological experiments.

### Measurement of cellular oxygen consumption rates

Oxygen consumption rates (OCR) were measured using XF24 extracellular flux analyzer (Seahorse, Agilent, Folsom, CA, USA) according to established protocols [23, 24, 25] and as we described [16, 21, 26]. B16F10 and YUMM1.7 cells were seeded in XF24 plates ([0.8‐1.2] × 10^4^/well), grown overnight, and treated as indicated. After treatments, culture media were replaced with unbuffered Dulbecco’s modified Eagle’s medium (Sigma, D5030) supplemented with 2 mm pyruvate/15 mm glucose/2 mm GlutaMAX (#35050, Invitrogen) adjusted to pH 7.4 and equilibrated in CO_2_‐free incubator at 37 °C. Three measurements at 5‐min intervals were recorded for each segment of assay. Sequential additions of mitochondrial effectors were via ports of XF24 cartridges; effectors concentrations were 2 µm oligomycin (O4876, Sigma), 2.5 μm carbonyl cyanide *p*‐trifluoromethoxyphenylhydrazone (FCCP) (C2920, Sigma), 50 mm 2‐deoxy‐d‐glucose (2DG) (D6134, Sigma), and 5.4 µm antimycin A (A8674, Sigma). The initial readings defined as baseline OCR were followed by measurements of ATP synthesis‐coupled OCR revealed by the addition of oligomycin. The difference between FCCP‐induced and baseline OCR was defined as spare respiratory capacity (SRC). The addition of 2DG demonstrated that in control cultures, inhibition of glycolytic pathway by 2DG leads to increases in OCR reflecting compensatory mitochondrial utilization of pyruvate and glutamine (supplied in XF24 assay medium). Measurements of OCR modulations induced by injections of effectors served to compare respiratory parameters between controls and test groups [23, 24]. Data are presented as mean ± SEM of four independent biological experiments. Each parameter measured in control cultures was assigned the value of 100%, and outcomes of treatments are given as percent change relative to each respective control.

### Statistical analysis

Data are provided as mean ± SEM calculated from 3 to 4 independent biological experiments, as stated. Two‐tailed Student’s *t*‐test was used to compare the means between groups. *P* value < 0.05 was considered statistically significant. megastat® software for Excel was used.

## Results

### Hypoxia upregulates glycolytic gene expression and increases extracellular lactate levels in B16F10 and YUMM1.7 melanoma cells

**Fig. 1:**
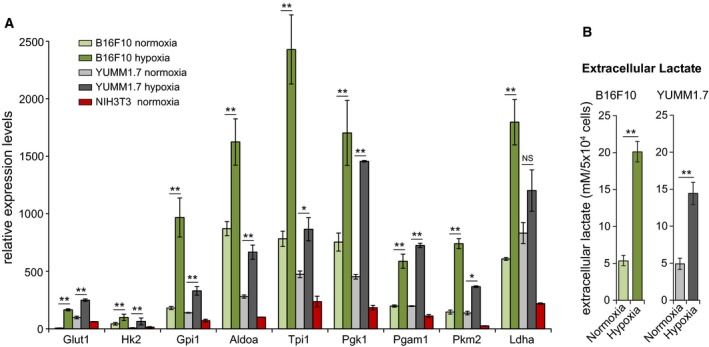
High baseline and upregulation by hypoxia of glycolytic gene expression in B16F10 and YUMM1.7 melanoma cells. (A) Glycolytic gene expression profiles in normoxia and hypoxia of B16F10 (green) and YUMM1.7 (gray) cells; mouse NIH3T3 fibroblast baseline expression pattern is shown for comparison (red). (B) Extracellular lactate levels in B16F10 and YUMM1.7 culture media measured following incubation under normoxic and hypoxic conditions. Values from 4 biological experiments were used to obtain mean ± SEM; two‐tailed t‐test was used. *P < 0.05 and **P < 0.01 versus respective mean value in normoxia.

Energy production in cancer cells involves aerobic glycolysis and mitochondrial respiration [1]. Compared to NIH3T3 embryonic fibroblasts, baseline expression of glycolytic genes in B16F10 and YUMM1.7 cells is markedly higher and sharply upregulated by hypoxia (Fig. 1A). Strongest upregulation was measured for genes encoding the rate controlling proteins, glucose transporter‐1, and hexokinase‐2 (Fig. 1A). The shift to glycolytic metabolism was reflected also in threefold and fourfold increases in extracellular lactate levels in YUMM1.7 and B16F10 cultures, respectively, after 14‐h hypoxia, compared to levels accumulated during the same period under normoxic culture conditions (Fig. 1B).

### Hypoxia‐associated reduction in mitochondrial contents of B16F10 and YUMM1.7 cells

**Fig. 2:**
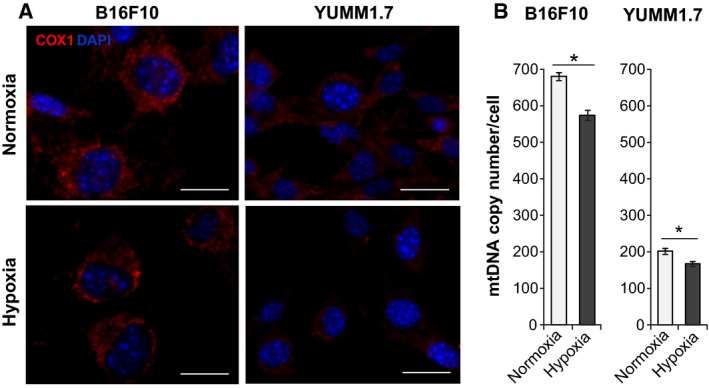
Cytochrome c oxidase subunit 1 (Cox1) immunoreactivity and mtDNA copy numbers decrease under hypoxic conditions in B16F10 and YUMM1.7 cells. (A) Representative images of Cox1 immunofluorescence patterns (red) observed under normoxic and hypoxic conditions; Intense Cox1 staining reflects high mitochondrial contents in B16F10 compared to YUMM1.7 cells. Staining intensity is reduced in hypoxia; nuclei stain blue with DAPI, scale bar = 20 µm. (B) RT‐qPCR analyses of mtDNA contents reveal reduction in mtDNA copy number under hypoxic conditions; data are presented as mean ± SEM copy number for 3‐4 experiments; two‐tailed t‐test was used. *indicates different from normoxia; *P* < 0.05.

Mitochondrial contents and distribution patterns in B16F10 and YUMM1.7 cells were evaluated by immunoreactivity of the mitochondria‐encoded cytochrome c oxidase subunit 1(Cox1) protein of respiratory complex IV. Markedly, stronger Cox1 immunoreactivity was observed in B16F10 when compared to YUMM1.7 cells (Fig. 2). Imaging also revealed differences in cell morphology including significantly larger nuclei and cell dimensions in B16F10 when compared to YUMM1.7 cells. Stronger Cox1 staining in B16F10 cells was consistent with RT‐qPCR results that revealed ~ 3‐fold higher mtDNA copy number in B16F10 compared to YUMM1.7 cells. Cox1 immunoreactivity and mtDNA contents decreased in both cell lines following hypoxic exposures (Fig. 2B).

### Hypoxia attenuates cisplatin‐ and doxorubicin‐induced proliferative arrest and cell death rates

**Fig. 3:**
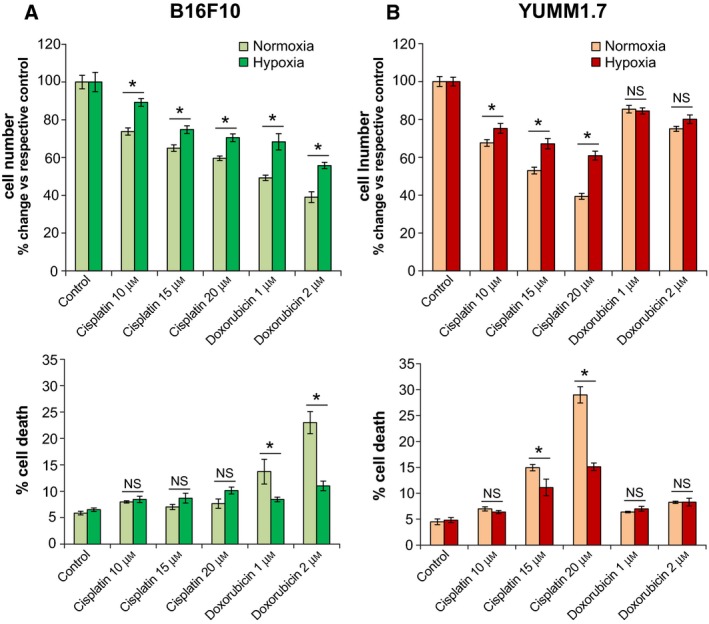
Differential decreases in proliferation and increases in cell death rates in B16F10 (A) and YUMM1.7 (B) cells incubated with cisplatin or doxorubicin under normoxic and hypoxic conditions. (Top) graphs show percent change in cell number relative to respective control following each treatment; data are presented as mean ± SEM of percent change averaged for 3 biological experiments; *Indicates different from mean ± SEM percent change in normoxia; P < 0.05. (Bottom) percent cell death in cultures challenged with cisplatin or doxorubicin under normoxic or hypoxic conditions; data are presented as mean ± SEM from 3 experiments; *Indicates different from percent cell death in normoxia, NS, not significant; *P* < 0.05. Two‐tailed *t*‐test was used.

To effectively compare the impact of cisplatin and doxorubicin on B16F10 and YUMM1.7 cells, treatment conditions were finely precalibrated to yield drug dose‐dependent simultaneously measurable effects, while avoiding high death rates in both cell lines. This was achieved in the course of 14‐h incubation with 10, 15, and 20 µm cisplatin or 1 and 2 µm doxorubicin under normoxic or hypoxic conditions. The above treatments elicited differential effects on cell proliferation and death rates, with B16f10 cells exhibiting greater sensitivity to doxorubicin and lesser sensitivity to cisplatin, when compared to YUMM1.7 cells subjected to identical treatments (Fig. 3). Importantly, the drug‐induced decreases in cell numbers versus respective controls were attenuated when exposures were done under hypoxic conditions. For B16F10 cells, the relative decline in cell number was between 25 and 60% under normoxic versus a 10–45% decrease under hypoxic conditions, with doxorubicin causing the sharpest declines (Fig. 3A, top). In contrast, YUMM1.7 cells were more sensitive to cisplatin with 30–60% decline in normoxia versus 25–40% in hypoxia (Fig. 3B, top). In addition to proliferative arrest, cisplatin and doxorubicin exposures differentially increased cell death rates, reaching in B16F10 14% and 23%, following normoxic exposures to 1 and 2 µm doxorubicin, respectively, but only 10% in hypoxia (Fig. 3A, bottom). In YUMM1.7 cells, following 15 and 20 µm cisplatin, death rates were 15 and 28%, respectively, and 12 and 15% under hypoxic conditions (Fig. 3B, bottom). Effects of doxorubicin in YUMM1.7 were modest with 7–8% cell death (compared to ~ 5% in nonexposed control cultures). Combined, the data show that DNA damaging drug‐induced decreases in melanoma cell numbers result from proliferative arrest and increases in cell death.

### Hypoxia protects B16F10 and YUMM1.7 cells from cisplatin and doxorubicin mitotoxicity

**Fig. 4:**
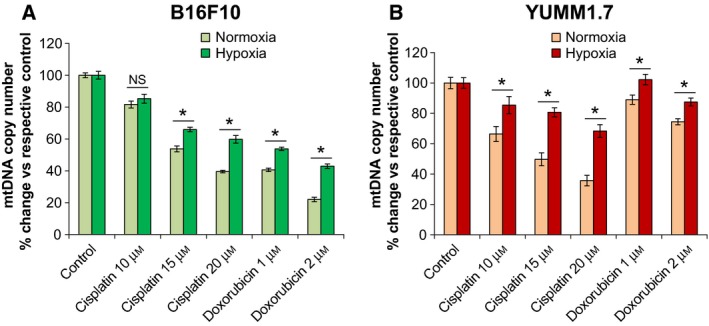
Differential decreases of mtDNA contents in B16F10 and YUMM1.7 cells following cisplatin and doxorubicin exposures under normoxic and hypoxic conditions. RT‐qPCR analyses of mtDNA copy number in B16F10 (A) and YUMM1.7 (B) cells; data are presented as mean ± SEM percent change in mtDNA copy number versus each respective control calculated from 3 biological experiments; two‐tailed t‐test was used, *indicates different from % change in normoxia, NS, not significant; *P* < 0.05.

**Fig. 5:**
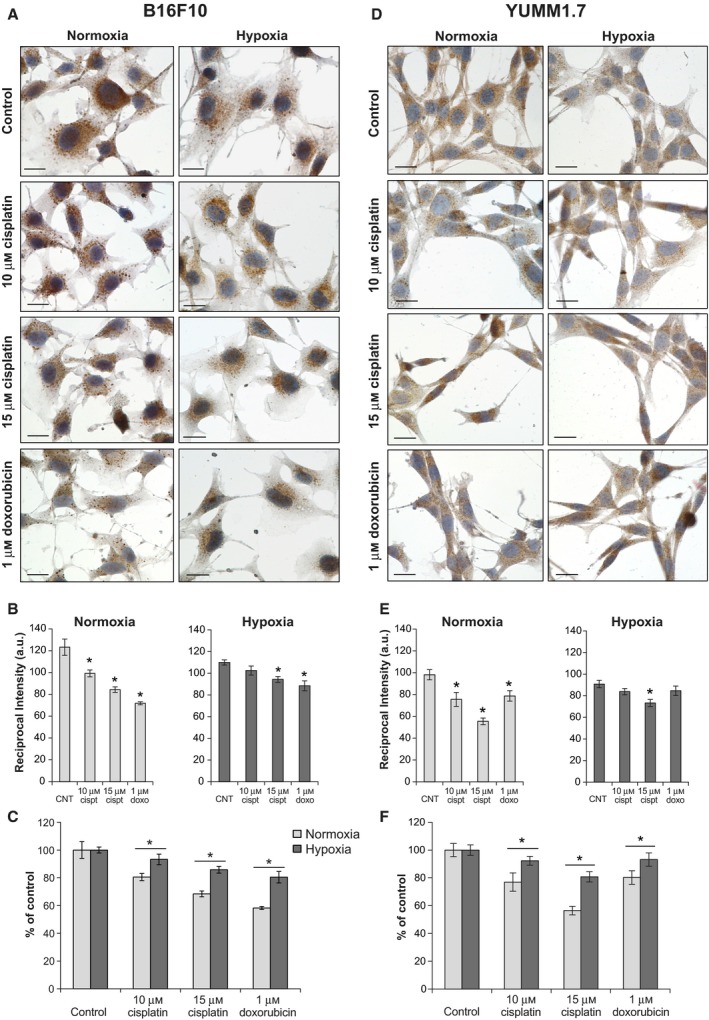
Immunoreactivity patterns of the mtDNA binding transcription factor A (Tfam) are differentially modified following exposures to cisplatin and doxorubicin. (A‐C) Representative images and quantitation of staining intensity of nontreated and drug‐exposed B16F10 cells show changes in Tfam intensity and distribution, with discrete aggregated pattern following cisplatin and doxorubicin exposures. Reduced staining intensity is seen following hypoxia. Hypoxia lessens drug‐induced Tfam aggregation when compared to normoxic conditions. (D–F) Representative images and quantitation of Tfam immunoreactivity in control and drug‐exposed YUMM1.7 cells. Staining patterns are differentially modified by cisplatin and doxorubicin with aggregation and spindle‐shaped cell morphology. Hypoxia attenuates the steep decline in staining intensity observed after normoxic drug exposures with reduced aggregation and fewer spindle‐shaped stressed cells present. Tfam is observed in brown, counterstained with hematoxylin, and captured with 60x oil objective; scale bar = 20 µm. Staining intensity quantification is shown in panels B and E; bars represent intensity values of TFAM immunoreactivity (a.u.). Asterisk indicates different from nonexposed normoxic or hypoxic control, respectively. In panels C and F, intensity values are normalized and presented as percent change relatively to each respective control; asterisk indicates different from the change measured for each drug exposure under normoxic condition. Data are graphed as mean ± SEM of 3 biological replicates per condition, *p < 0.05. Two‐tailed *t*‐test was used.

The larger B16F10 cells have ~ 3‐fold higher mtDNA contents and markedly higher Cox1 immunoreactivity compared to YUMM1.7 cells (Fig. 2). Since DNA damaging drugs readily damage unprotected mtDNA, we asked whether cisplatin or doxorubicin exposures might lead to mtDNA depletion and detected dose‐dependent decreases in mtDNA copy number in both, B16F10 and YUMM1.7 cells (Fig. 4). MtDNA contents declined sharply in B16F10 cells, with ~ 80% and ~ 50% decreases with 2 µm doxorubicin, in normoxia and hypoxia, respectively. In YUMM1.7 cells, sharp 65% and 35% decline was measured following 20 µm cisplatin exposure, in normoxia and hypoxia, respectively. Changes in mtDNA contents in drug‐exposed B16F10 and YUMM1.7 cells were accompanied by changes in immunoreactivity patterns of the mitochondrial transcription factor A (Tfam), which coats mtDNA and is the main component of mtDNA nucleoids [27]. Tfam immunoreactivity was assessed in B16F10 and YUMM1.7 cells in the absence/presence of cisplatin or doxorubicin under normoxic and hypoxic conditions (Fig. 5). Consistent with reduced Cox1 immunoreactivity and mtDNA contents under hypoxic conditions, Tfam immunoreactivity also decreased following hypoxia. Cisplatin and doxorubicin exposures exerted differential effects on Tfam distribution. In B16F10 cells, exposures to cisplatin under normoxic conditions resulted in aggregated Tfam immunoreactivity pattern and aggregated weakened staining with doxorubicin. Following hypoxia, Tfam intensity in B16F10 cells was reduced, whereas drug exposures under hypoxic conditions showed lesser aggregation, suggestive of diminished mitotoxicity (Fig. 5A). In YUMM1.7 cells, baseline Tfam immunoreactivity was markedly weaker (Fig. 5D), consistent with lower mtDNA contents. After normoxic incubation with 15 µm cisplatin, Tfam immunoreactivity was reduced and cells acquired spindle‐like morphology (Fig. 5D). Interestingly, spindle‐like cell morphology was reported in regressing tumors generated with YUMMER1.7 cells, which were derived from the YUMM1.7 cell line exposed to UVB [28]. Perturbations in Tfam immunoreactivity patterns were attenuated when B16F10 and YUMM1.7 cells were subjected to identical treatments under hypoxic conditions (right panels), suggesting that less active mitochondria are less susceptible to DNA damaging drugs. Quantification of Tfam immunoreactivity under the different treatment conditions revealed markedly attenuated decline in staining intensity in response to drug exposures under hypoxic compared to normoxic conditions in both, B16F10 (Fig. 5B,C) and YUMM1.7 (Fig. 5E, F) cell lines. Interestingly, the protective effects of hypoxia were most pronounced when the impact of drug was the strongest, that is, attenuation of the decline in Tfam staining intensity caused by 1 µm doxorubicin in the case of B16F10 and that caused by 15 µm cisplatin in the case of YUMM1.7.

The protective effects of hypoxia on functional parameters of B16F10 and YUMM1.7 cells were further substantiated by the shift in cisplatin and doxorubicin IC50 values obtained under hypoxic versus normoxic conditions. IC50 values were calculated for cell proliferation and cell death as well as depletion of mtDNA [Table 2]. Collectively, IC50 values corroborate the observed differential vulnerability of these cell lines to cisplatin and doxorubicin and confirm that mtDNA is a particularly vulnerable target that is shielded under hypoxic conditions.

**Table 2:** Hypoxia alters IC50 Values of cisplatin and doxorubicin in B16F10 and YUMM1.7 melanoma cells

IC50 μM^a^	B16F10		YUMM1.7	
	Normoxia	Hypoxia	Normoxia	Hypoxia
Cell Proliferation				
Cisplatin	30	33	16	31
Doxorubicin	1.16	2.34	5.51	10.58
Cell death				
Cisplatin	> 50	> 50	28	50
Doxorubicin	17	37	> 50	> 50
mtDNA copy number				
Cisplatin	18	25	15	34
Doxorubicin	0.69	1.42	4.14	6.85

^a^ Values are based on 14‐h drug exposures as described in Figs 3 and 4.

### Hypoxia attenuates cisplatin‐ and doxorubicin‐induced respiratory dysfunction

**Fig. 6:**
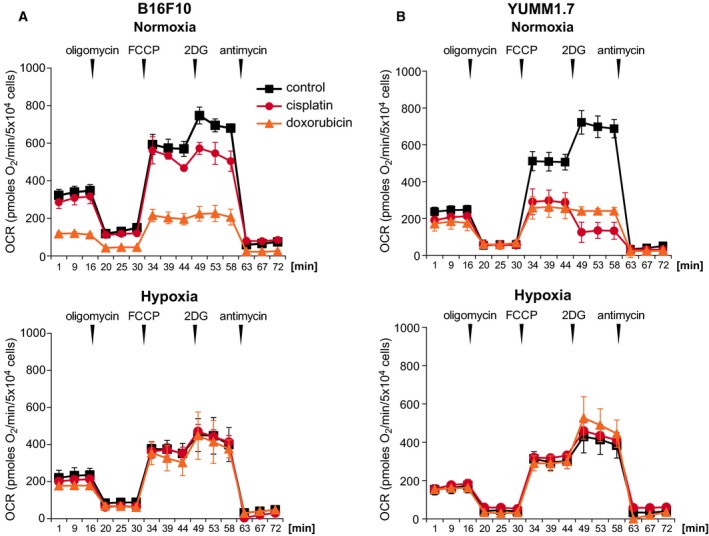
Hypoxia protects B16F10 and YUMM1.7 cells from cisplatin and doxorubicin‐induced respiratory compromise. Respiratory assays were done on XF24 extracellular flux analyzer. Post‐treatment respiratory profiles of (A) B16F10 and (B) YUMM1.7 cells following incubation with vehicle, cisplatin, or doxorubicin under normoxic (top) and hypoxic (bottom) conditions. Additions of effectors are indicated by vertical arrowheads. The first segment of assay measures baseline respiration followed by the addition of oligomycin that reveals the portion of OCR coupled to ATP synthesis, followed by measurements of FCCP‐induced OCR and subsequent assessments of mitochondrial compromise revealed by the addition of 2DG that blocks glycolytic metabolism and stimulates compensatory mitochondrial oxygen consumption. Values are presented as mean ± SEM OCR for 4 biological experiments.

Next, we asked whether mitochondrial respiration in B16F10 and YUMM1.7 cells is impaired by drug exposures. Respiratory parameters were assessed using the extracellular flux analyzer, XF24, which measures changes in cellular OCR (Fig. 6). Cultures challenged with drugs under normoxic and hypoxic conditions were processed in parallel and XF24‐implemented sequential additions of mitochondrial effectors, oligomycin, FCCP, 2‐deoxyglucose (2DG), and antimycin A, served to determine the treatments signatures on respiratory parameters, as we previously described [16, 26]. Under normoxic conditions, baseline OCR in YUMM1.7 was ~ 30% lower compared to B16F10 cells. The increase in OCR upon addition of FCCP, which reflects cell capacity for substrate oxidation, was ~ 2‐fold higher than baseline in B16F10 and ~ 2.4‐fold higher in YUMM1.7 cells. The difference between the FCCP‐induced and baseline OCR, which is defined as spare respiratory capacity (SRC), was 100% in B16F10 and 140% in YUMM1.7 cells. Subsequent addition of 2DG, an inhibitor of the glycolytic pathway‐controlling enzyme, hexokinase 2, resulted in a further ~ 30% increase in OCR in both cell lines, reflective of increases in mitochondrial respiration fueled by glutamine and pyruvate supplied in XF24 assay medium, which are utilized when glycolysis is halted. Cisplatin and doxorubicin exposures differentially affected mitochondrial respiration. In B16F10 cells under normoxic conditions, cisplatin (15 µm) had a modest effect, which was mainly reflected in inability to increase mitochondrial OCR following the addition of 2DG. In contrast, 1 µm doxorubicin had marked effect on respiratory parameters, as reflected in drastic decreases in baseline OCR and inability to respond to 2DG (Fig. 6A, top). Conversely, while in YUMM1.7 cells baseline respiration was only marginally perturbed, cisplatin and doxorubicin severely affected the spare respiratory capacity (Fig. 6B, top). Moreover, while similar to B16F10 cells, doxorubicin‐exposed YUMM1.7 cells were unable to respond to 2DG, cisplatin‐exposed YUMM1.7 cells showed an additional 50% reduction in OCR, suggesting that due to severe cisplatin mitotoxicity in YUMM1.7 cells, more than 50% of consumed oxygen fuels glycolytic metabolism.

Parallel analyses done under hypoxic conditions (Fig. 6, bottom) revealed that baseline OCR in B16F10 and YUMM1.7 cells was lowered by ~ 35% and 25%, respectively, and spare respiratory capacity was reduced relatively to normoxia. However, when cisplatin and doxorubicin were added under hypoxic conditions, respiratory parameters were significantly less affected compared to drug exposures under normoxic conditions, with respiratory profiles similar to those elicited by hypoxia alone (Fig. 6, bottom). In B16F10, baseline OCR was not reduced by drugs relatively to OCR measured following hypoxia alone, and unlike in the case of normoxia, the cells retained their ability to respond to both, FCCP and 2DG, albeit to a lesser extent (Fig. 6A, bottom). In YUMM1.7 cells, the protective effects of hypoxia were more pronounced, in that unlike under normoxic conditions, YUMM1.7 cells mitochondria retained measurable ability to respond to both, FCCP and 2DG following cisplatin exposure (Fig. 6B, bottom).

## Discussion

Cancer cells utilize mitochondrial and glycolytic metabolism for energy production and rely on metabolic plasticity to sustain vigorous growth, adapt to changing conditions, and evade cytotoxic threats [29, 30, 31, 32, 33]. Here, we demonstrate that B16F10 and YUMM1.7 melanoma cells upregulate glycolytic metabolism under hypoxic conditions and avert cisplatin and doxorubicin‐induced mitochondrial dysfunction. In line with ample evidence for the involvement of aerobic glycolysis in cancer cell energy metabolism [34, 35], B16F10 and YUMM1.7 cells exhibit high baseline glycolytic gene expression with strong upregulation by hypoxia, especially of genes encoding the glycolytic pathway‐controlling proteins, glucose transporter 1, which facilitates glucose uptake [36] and hexokinase 2 that catalyzes the first and rate limiting step of glycolysis [37]. In both cell lines, hypoxia‐induced upregulation of glycolytic genes is accompanied by robust increases in extracellular lactate levels (Fig. 1B) and concomitant decreases in mitochondrial contents.

Notwithstanding these similarities, B16F10 and YUMM1.7 cells differ in their ability to withstand cisplatin and doxorubicin exposures. Proliferation of B16F10 cells is reduced by doxorubicin to a greater extent than by cisplatin; conversely, under identical conditions, YUMM1.7 cell proliferation is reduced to a greater extent by cisplatin than by doxorubicin. Additionally, marked increases in B16F10 cell death rates are observed following doxorubicin, whereas in YUMM1.7 cells, death rates increase with cisplatin but not with doxorubicin exposures. Likewise, in B16F10 cells, mtDNA is depleted to a greater extent by doxorubicin, while in YUMM1.7 cells, greater depletion occurs with cisplatin and is accompanied by distinctive changes in mtDNA distribution as reflected in immunoreactivity patterns of the mtDNA binding protein, Tfam. The protective effects of hypoxia in both cell lines are reflected in the marked differences in cisplatin and doxorubicin IC50 values attained in assays carried out under normoxic and hypoxic conditions. IC50 values also substantiate the observed differential sensitivities of B16F10 and YUMM1.7 cells to these drugs.

Importantly, differences in vulnerabilities are manifested also in differential effects on mitochondrial respiratory parameters revealed as changes in OCR. Exposure of B16F10 cells to doxorubicin resulted in reduced baseline OCR, reduced spare respiratory capacity, and inability to elevate OCR following the addition of 2DG that inhibits hexokinase 2, a key regulatory enzyme which catalyzes the first step of glycolysis. By inhibiting hexokinase, 2DG suppresses glycolytic metabolism and reduces the contribution of pyruvate to mitochondrial oxidative phosphorylation. Under normal conditions, functional mitochondria compensate for diminished glycolysis by increasing mitochondrial utilization of other nutrients such as glutamine. However, mitochondria that have been compromised by drug exposures were unable to efficiently utilize glutamine, as reflected in inability to increase OCR in response to 2DG, as observed in B16F10 cells exposed to doxorubicin and to some degree also to cisplatin. Interestingly, YUMM1.7 cells were more affected and unable to increase mitochondrial OCR in response to 2DG after incubation with doxorubicin, and more so in the case of cisplatin, resulting in a further decrease in OCR, indicating that due to severe cisplatin mitotoxicity in YUMM1.7 cells, a substantial portion of oxygen consumed under normoxic conditions in the presence of cisplatin, fuels aerobic glycolysis.

Cisplatin and doxorubicin belong to different classes of DNA damaging drugs that induce damage by different mechanisms [2, 7]. Cisplatin crosslinks in nuclear DNA are repaired via NER pathway that is absent from mitochondria [4], where cisplatin crosslinks persist and compromise respiring mitochondria, while exerting lesser effect on the less metabolically active mitochondria under hypoxic conditions [38]. It is plausible therefore that some cancer cells that more readily shift to aerobic glycolysis under normoxic conditions might better withstand cisplatin exposures. This could be the case with B16F10 cells that exhibit lesser sensitivity to cisplatin. Doxorubicin, on the other hand, intercalates between DNA base pairs and inhibits Top2 by stabilizing toxic Top2 complexes with DNA ends leading to nuclear and mitochondrial dysfunction [7, 39]. Interestingly, Top1 has been implicated in the release of doxorubicin from toxic complexes [40], suggesting that cells endowed with high Top1 activity might be able to better withstand doxorubicin‐induced damage. It is unknown if highly active Top1 variants exist in certain cancer cells and help evade the tumor growth‐curtailing the effects of doxorubicin.

Importantly, both cell lines better withstand exposures to cisplatin and doxorubicin under hypoxic conditions. Hypoxia might mitigate detrimental effects of DNA damaging drugs by different mechanisms, including upregulation of hypoxia‐inducible factor‐1 (Hif‐1) and Hif‐1‐mediated modulation of gene expression and signaling that promote resistance to chemotherapeutic drugs [41]. Hypoxia may transiently halt cell division and DNA replication, thereby limiting drug access to the more tightly packaged nonreplicating DNA. Since under hypoxic conditions cancer cells shift to glycolytic metabolism, the subsequently less active mitochondria [38], may slow mtDNA replication and limit mtDNA damage. Moreover, reduced mitochondrial contents following oxygen deprivation might diminish ROS levels and allow cells to better withstand challenging conditions. Taken together, our findings reveal divergent vulnerabilities of B16F10 and Yumm1.7 melanoma cells to DNA damaging drugs and suggest that hypoxic environment might enable distinct melanoma tumors to evade mitotoxic and cytotoxic effects of chemotherapy.

Presently, despite continually expanding cancer treatment options, it remains difficult to predict which tumors might respond to which treatments [42, 43]. Our data underscore the need to uncover genetic weaknesses that might render tumors derived from the same tissue differentially responsive to particular therapies and reveal mechanisms that tumors might utilize for drug evasion.

## Conflict of interest

The authors declare no conflict of interest.

## Author contributions

EWE and DST conceived and developed the study. MZ and FMG performed the experiments and data analyses. EWE and MZ developed the experiments and interpreted data, and EWE wrote the manuscript.
